# NRF1 and NRF2 Expression in Preeclamptic Placentas: A Comparative Observational Study

**DOI:** 10.3390/life16010089

**Published:** 2026-01-07

**Authors:** Şehmus Kaplan, Uğur Karabat, Muhyiddin Sancar, Fırat Aşır, Elif Ağaçayak

**Affiliations:** 1 Department of Histology and Embryology, Medical Faculty, Dicle University, 21280 Sur, Diyarbakır, Turkey; 2 Department of Gynecology and Obstetrics, Medical Faculty, Dicle University, 21280 Sur, Diyarbakır, Turkey

**Keywords:** preeclampsia, placenta, NRF1, NRF2, oxidative stress, bioinformatics

## Abstract

Background: Preeclampsia (PE) is a hypertensive disorder of pregnancy associated with oxidative stress and mitochondrial dysfunction. NRF1 and NRF2 are transcription factors that regulate mitochondrial activity and antioxidant defense. This study investigated their expression patterns in placentas from preeclamptic and severe preeclamptic pregnancies by immunohistochemical and bioinformatical methods. Methods: Placentas from 40 healthy controls, 40 PE, and 40 sPE patients were analyzed by histological and immunohistochemical techniques. Protein–protein interaction networks for NRF1, NRF2, and PE-related proteins were constructed using Search Tool for the Retrieval of Interacting Genes/Proteins (STRING) and Cytoscape software, followed by Kyoto Encyclopedia of Genes and Genomes (KEGG) pathway enrichment analysis performed via ShinyGO, with significance set at false discovery rate (FDR) < 0.05. Results: NRF1 expression was significantly decreased in PE and sPE groups compared to controls, with notably negative staining in syncytial knots and fibrinoid areas. Conversely, NRF2 expression significantly increased, showing intense positivity in syncytiotrophoblasts, stromal cells, and vascular structures. Pathway analysis revealed that decreased NRF1 expression was associated with glutathione metabolism, hypoxia inducible factor-1 (HIF-1) signaling, and AMP-Activated Protein Kinase (AMPK) signaling pathways. Increased NRF2 expression was associated predominantly with inflammatory and immune response pathways, including AGE-RAGE signaling and pathogen–response pathways. Conclusions: Differential expressions of NRF1 and NRF2 in preeclamptic placentas reflect distinct yet interconnected responses to oxidative stress and inflammation. These transcription factors have potential clinical relevance as biomarkers for PE severity assessment and as targets for future therapeutic interventions.

## 1. Introduction

Preeclampsia (PE) is a pregnancy-specific disorder that affects 3–5% of pregnancies worldwide and remains a major cause of maternal and neonatal morbidity and mortality [1]. It is clinically characterized by new-onset hypertension and proteinuria after 20 weeks of gestation and pathophysiologically by abnormal placental development and systemic endothelial dysfunction [2]. Despite extensive investigation, the molecular mechanisms underlying PE remain only partially understood [3].

A growing body of evidence indicates that oxidative stress and mitochondrial dysfunction play central roles in the pathogenesis of PE [4,5]. Excessive production of reactive oxygen species within the placenta contributes to trophoblastic apoptosis, vascular injury, and impaired perfusion [6,7]. Among the transcription factors that regulate cellular responses to oxidative stress, Nuclear Respiratory Factor 1 (NRF1) and Nuclear Factor Erythroid 2-Related Factor 2 (NRF2) are of particular importance. NRF1 controls mitochondrial biogenesis and energy metabolism, whereas NRF2 orchestrates antioxidant defense mechanisms by inducing genes involved in detoxification and redox balance [8,9].

Previous studies have suggested that dysregulation of NRF1 and NRF2 may contribute to placental oxidative injury in preeclampsia [10]. Reduced NRF1 activity has been linked to impaired mitochondrial function and defective trophoblast differentiation [11], while enhanced NRF2 activation may represent a compensatory response to excessive oxidative stress [12,13]. However, their coordinated behavior and the specific molecular pathways involved in preeclamptic placentas remain unclear.

Therefore, this study aimed to investigate the expression patterns of NRF1 and NRF2 in placental tissues from preeclamptic and severe preeclamptic pregnancies and to identify the molecular pathways associated with their dysregulation using immunohistochemical and bioinformatic analyses. Clarifying the relationship between these two transcription factors may help to elucidate oxidative stress-related mechanisms and identify potential biomarkers or therapeutic targets for preeclampsia.

## 2. Materials and Methods

### 2.1. Ethical Approval and Study Design

This study was conducted in accordance with the Declaration of Helsinki and was approved by the Non-Interventional Clinical Research Ethics Committee of Dicle University Faculty of Medicine (approval date: 28 February 2023, number: 2023/74). Written informed consent was obtained from all participants prior to inclusion in the study. The study was designed as an observational, comparative research design. It integrated clinical data, histological analysis, immunohistochemical staining, and bioinformatics approaches to investigate the role of NRF1 and NRF2 in PE pathophysiology. The workflow included patient selection, sample collection, histopathological and immunohistochemical assessments, and computational pathway analysis to identify molecular mechanisms associated with PE.

### 2.2. Placenta Collection

To ensure consistency and validity of the study’s classification of patient groups, the diagnostic criteria for PE and sPE were categorized in accordance with The American College of Obstetricians and Gynecologists (ACOG) criteria [14]. The PE group included women diagnosed with PE with BP ≥ 140/90 mmHg and proteinuria ≥ 300 mg/24 h or signs of end-organ dysfunction. The sPE group included patients with severe hypertension (blood pressure ≥ 160/110 mmHg), thrombocytopenia, renal insufficiency, impaired liver function, pulmonary edema, or neurological complications. The control group consisted of healthy pregnant women without a history of hypertension, diabetes, renal, or other systemic diseases. Patients with multiple pregnancies, fetal anomalies, chronic diseases, or a history of placental abruption were excluded from the study. The study included pregnant women aged 18–49 years who delivered at Department of Gynecology and Obstetrics, Dicle University Faculty of Medicine. The patients were informed about the study and their informed consent was obtained. Additional exclusion criteria included chronic hypertension, diabetes mellitus, autoimmune diseases, renal disorders, infectious diseases, multiple pregnancies, fetal congenital anomalies, placental abruption, and active labor at the time of delivery.

### 2.3. Placenta Tissue Preparation

Placentas from 40 healthy control pregnant women, 40 placentas from women with preeclampsia, and 40 placentas from women with severe preeclampsia were collected immediately after delivery. Samples were taken only from the fetal side after carefully removing amniotic membranes. Infarcted and necrotic areas were avoided to maintain histological integrity. The samples were rinsed with phosphate-buffered saline (PBS) before fixation to remove any residual blood and contaminants. Each placenta yielded approximately 5–7 sections, with at least three sections used for immunohistochemical staining. The control group included both cesarean section (CS) and mostly normal spontaneous delivery (NSD) cases, while PE and sPE groups consisted primarily of CS deliveries due to medical indications. Active labors occurring at the delivery time were not included in the study to eliminate potential confounding factors related to labor-induced physiological changes. Placenta samples were fixed in %10 formaldehyde solution for 24 h, followed by ascending ethanol series (50%, 70%, 80%, 90%, 96%, and absolute ethyl alcohol) and kept in xylene solution. Samples were embedded in paraffin blocks and 4–6 μm thick sections were taken with a microtome (catalog no: Leica RM2265, Leica Camera AG, Wetzlar, Germany) [15].

### 2.4. Immunohistochemical Staining

Sections were washed in phosphate buffer solution (PBS) for 3 × 5 min. After epitope retrieval with ethyl diamine tetraacetic acid (EDTA) solution (pH: 8.0, catalogue no: ab93680, Abcam, Cambridge, MA, USA), sections were treated with hydrogen peroxide solution (catalogue no: TA-015-HP, ThermoFischer, Fremont, CA, USA) for 20 min. Nonspecific staining was blocked with blocking solution (catalog no: TA-015-UB, ThermoFischer, Fremont, CA, USA). Primary antibodies NRF1 and NRF2 (catalog no: ab175932 and ab137550, respectively, Abcam, Heidelberg, Germany, dilution ratio: 1/100) was dipped onto the tissues and left overnight at +4 °C. Following biotinylated secondary antibody (catalog no: TP-015-BN, ThermoFischer, Fremont, CA, USA), biotin–streptavidin complex was formed (catalog no: TS-015-HR, ThermoFischer, Fremont, CA, USA). Diaminobenzidine (DAB) (catalog no: TA-001-HCX, ThermoFischer, Fremont, CA, USA) was used as a chromogen. Gill III Hematoxylin staining was used as a counter stain. Sections were quickly passed through an increasing ethanol series, cleared in xylene and mounted with mounting medium (Entellan^®^, Merck, Darmstadt, Germany) and visualized with a Zeiss Imager A2 light microscope (Carl Zeiss Microscopy, LLC, White Plains, NY, USA) [16].

### 2.5. Semi-Quantitative Analysis

For the semiquantitative assessment of NRF1 and NRF2 expression, staining intensity was measured using ImageJ software (version 1.53, http://imagej.nih.gov/ij (accessed on 15 March 2025)), according to the protocol described by Crowe et al. [17]. Ten microscopic fields per sample were analyzed in each group, and the quantification results were documented. Brown coloration indicated positive antibody staining, whereas blue represented negative staining. Signal intensity (expression) within each area was obtained by dividing the stained antibody area by the total examined area. For each sample, the ratio of positively stained area relative to the total area was calculated across ten fields, and subsequently, a mean value for each group was determined. These mean values were then used for semiquantitative immunohistochemical scoring.

### 2.6. Functional Enrichment Analysis of NRF1- and NRF2-Associated Protein Targets in Preeclampsia

To investigate the pathways affected by NRF1- and NRF2-associated protein targets, which exhibit decreased and increased expression in preeclampsia (PE), respectively, Kyoto Encyclopedia of Genes and Genomes (KEGG) enrichment analysis was performed using ShinyGO (v0.81) [18]. Protein–protein interaction (PPI) networks for NRF1 and NRF2 were constructed using Cytoscape (v3.10.3), with 100 additional interactors. The PE-associated PPI network was generated using the Search Tool for the Retrieval of Interacting Genes/Proteins (STRING) database with 300 additional interactors and imported into Cytoscape. All PPI networks were created at a medium confidence level (0.400). Subsequently, shared proteins between NRF1- and NRF2-associated networks and the PE-specific network were identified. Furthermore, the Maximal Clique Centrality (MCC) algorithm was used to prioritize key proteins in the network, improving the identification of essential regulatory components [19]. KEGG pathway enrichment analysis was then conducted on these shared proteins, with significance defined as a False Discovery Rate (FDR) < 0.05. The top 10 significant pathways were ranked based on fold enrichment values [20].

### 2.7. Statistical Analysis

The statistical analysis was performed using IBM SPSS 25.0 software (IBM, Armonk, NY, USA). Data distribution was evaluated with the Shapiro–Wilk test. Normal distributed data was shown as mean ± standard deviation. Multiple comparisons were tested with ANOVA, followed by Tukey test for binary comparisons. Significance was considered for *p* values < 0.05 [21].

## 3. Results

### 3.1. Demographics Properties of Patients

Demographic and clinical features of the patients were shown in Table 1. A statistically significant was observed in systolic/diastolic blood pressure, 24 h proteinuria, platelet count, gestational week at birth, fetal birth, and APGAR scores between control and preeclamptic groups as characteristics of preeclampsia.

### 3.2. NRF1 Expression Was Downregulated in PE

**Figure 1:**
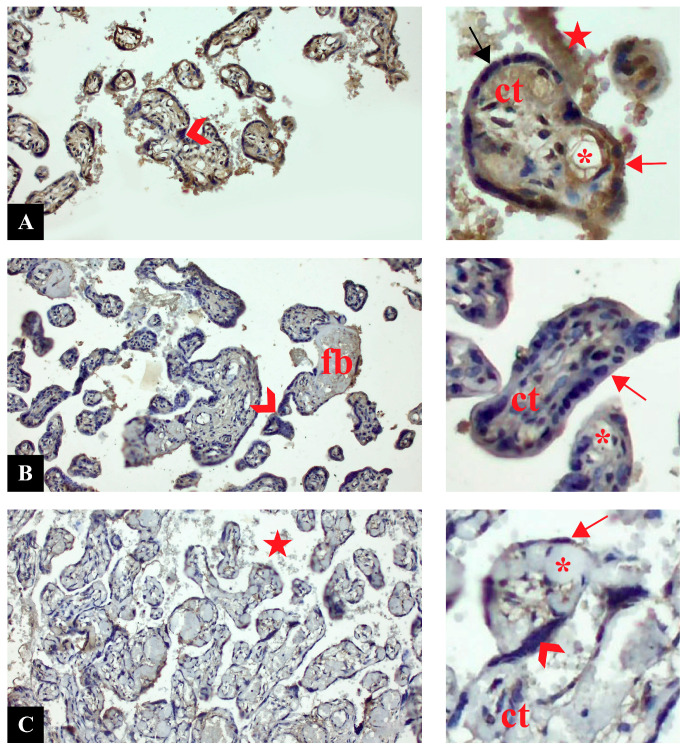
Cross sections of placental tissue. (**A**): Control. (**B**): Preeclampsia. (**C**): Preeclampsia with severe features. NRF1 immunostaining. Insets are higher magnification of sections. Arrow: syncytiotrophoblast cell (red: positive, black: negative), arrowhead: syncytial knot, ct: connective tissue, fb: fibrin deposition, star: intervillous area, *: capillary (Magnification: 20×, scale bar: 50 µm).

Cross sections of placental tissues belonging to control, PE, and PE with severe features groups with NRF1 immune staining are shown in Figure 1. In the control group, a generally moderate positive expression was observed in terms of NRF1 expression. While strong expression was observed in some syncytiotrophoblasts, negative expression was observed in some areas. NRF1 expression in syncytial nodes was generally negative. NRF1 immune reaction was observed as positive in the connective tissue and capillaries of placental villi. NRF1 expression was also observed in the cells of the intervillous area (Figure 1A). In the preeclampsia group, NRF1 expression was decreased compared to the control group. In placental structures, NRF1 immune reaction was generally observed as negative. Negative NRF1 expression was clearly observed in trophoblastic cells and syncytial nodes. Positive expression was observed in fibrinoid areas. NRF1 expression was observed as negative in connective tissue cells in placental villi and fetal capillaries (Figure 1B). NRF1 expression in the severe preeclampsia group was generally negative, similar to that in the preeclampsia group. Negative NRF1 expression was observed in syncytiotrophoblast cells and syncytial nodes. Mildly positive NRF1 expression was observed in fibrinoid areas. Negative expression was observed in vascular vessels. NRF1 expression was observed positive in some cells in the intervillous area (Figure 1C).

### 3.3. NRF2 Expression Was Upregulated in PE

**Figure 2:**
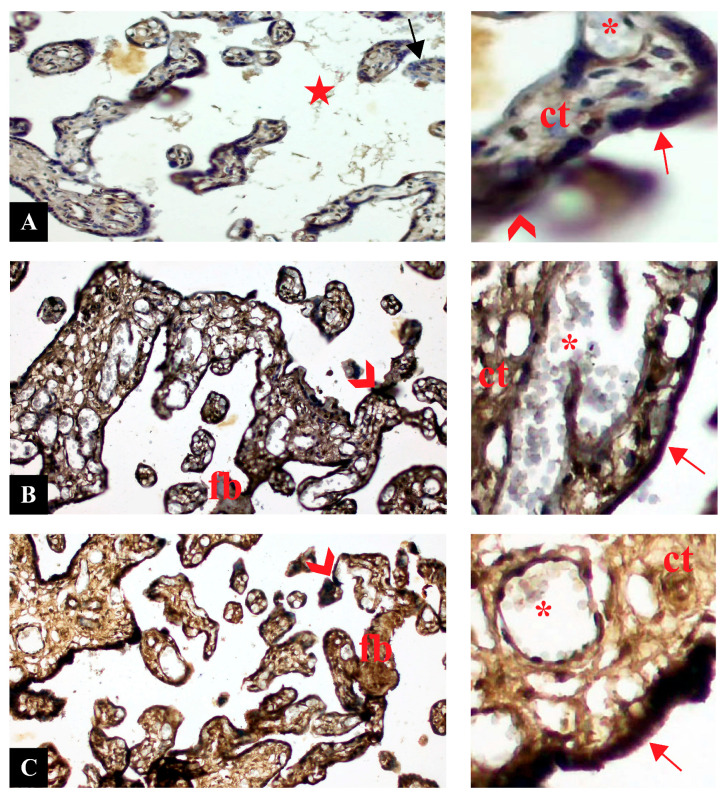
Cross sections of placental tissue. (**A**): Control. (**B**): Preeclampsia. (**C**): Preeclampsia with severe features. NRF2 immunostaining. Insets are higher magnification of sections. Arrow: syncytiotrophoblast cell (red: positive, black: negative), arrowhead: syncytial knot, ct: connective tissue, fb: fibrin deposition, star: intervillous area, *: capillary (Magnification: 20×, scale bar: 50 µm).

Cross sections of placental tissues belonging to control, PE, and PE with severe features groups with NRF2 immune staining are shown in Figure 2. NRF2 expression was generally observed at a moderate level in placental sections of the control group. NRF2 immune reaction was observed as positive in syncytiotrophoblasts, connective tissue cells and fetal capillaries of placental villi. Negative NRF2 expression was observed in the trophoblastic layer of some placental villi. Positive NRF2 expression was observed in syncytial nodes (Figure 2A). NRF2 expression in the preeclampsia group showed a significant increase compared to the control group. Increased NRF2 expression was observed in all placental structures. Intense expression was observed in syncytiotrophoblast cells and syncytial nodes. Strong positive NRF2 immune reaction was observed in connective tissue areas and fetal capillary endothelium. NRF2 expression was relatively less intense in fibrinoid areas (Figure 2B). NRF2 expression in the severe preeclampsia group was similarly positive compared to the preeclampsia group. NRF2 expression was significantly stronger compared to the control group. Intense NRF2 immune reaction was observed in trophoblastic cells, syncytial nodes, and fibrin accumulation. NRF2 expression was intense in vascular endothelial cells and connective tissue cells (Figure 2C).

### 3.4. Semiquantitative Measurement of NRF1 and NRF2 Expression

**Figure 3:**
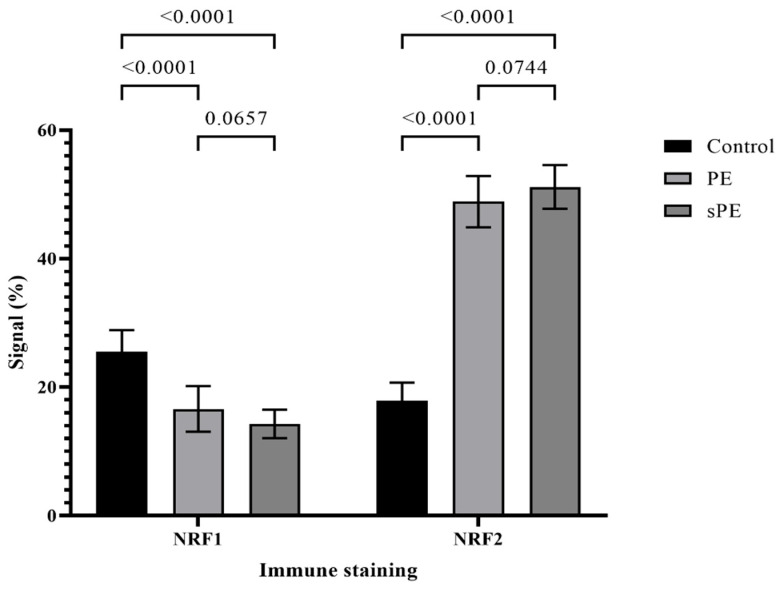
Graphical illustration of signal intensity (expression) of NRF1 and NRF2 proteins per group.

NRF1 and NRF2 expression was analyzed and semi-quantitatively measured by ImageJ software (Figure 3). A statistical significance was recorded in preeclamptic groups compared to control groups both in NRF1 and NRF2 expression. Downregulation of NRF1 and upregulation of NRF2 was evident in PE and sPE compared to control group. No significance was recorded between preeclamptic groups.

### 3.5. NRF1 and NRF2 Shared Pathways in Preeclampsia

**Figure 4:**
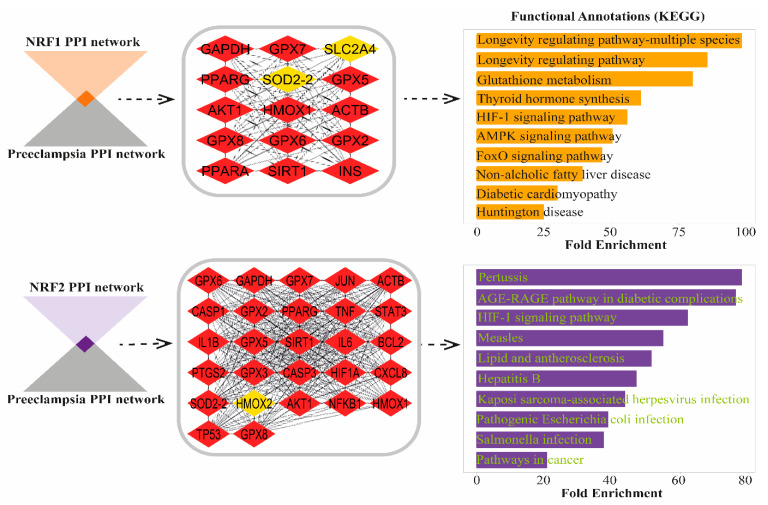
Pathway enrichment analysis of shared proteins between NRF1, NRF2, and PE. PPI networks for NRF1 and NRF2, showing shared targets with PE. The MCC algorithm in Cytoscape was used for protein sorting, where red nodes represent the most essential proteins with the highest centrality scores, while yellow nodes indicate proteins of lower significance. The bar graphs represent the top 10 enriched KEGG pathways for both NRF1 (**above**)- and NRF2 (**below**)-associated proteins in PE.

The pathway analysis identified 15 common proteins between NRF1 and PE, and 27 common proteins between NRF2 and PE (Figure 4). MCC-based analysis of the NRF1- and NRF2-associated PPI network revealed that most proteins play central regulatory roles, with several high-ranked nodes displaying strong interconnectivity. Functional enrichment analysis of proteins shared between NRF1 and PE, which showed decreased expression in PE groups, revealed statistically significant annotations (FDR < 0.05) for the following pathways: longevity regulating pathway-multiple species, longevity regulating pathway, glutathione metabolism, thyroid hormone synthesis, hypoxia-inducible factor 1 (HIF-1) signaling pathway, AMPK signaling pathway, FoxO signaling pathway, non-alcoholic fatty liver disease, diabetic cardiomyopathy, and Huntington disease. For NRF2, which exhibited increased expression in PE groups, functional enrichment analysis of shared targets with PE identified significant pathways (FDR < 0.05) including pertussis, Advanced Glycation End-products (AGE)–Receptor for Advanced Glycation End-products (RAGE) (AGE-RAGE) signaling pathway in diabetic complications, HIF-1 signaling pathway, measles, lipid and atherosclerosis, hepatitis B, kaposi sarcoma-associated herpesvirus infection, pathogenic Escherichia coli infection, Salmonella infection, and pathways in cancer.

## 4. Discussion

Preeclampsia (PE) is a complex, multisystem disorder characterized by placental ischemia, endothelial dysfunction, and oxidative stress [22,23]. In the present study, we demonstrated that NRF1 expression was significantly downregulated, whereas NRF2 expression was markedly upregulated in placentas from both preeclamptic and severe preeclamptic pregnancies. These findings indicate a differential but complementary role of these transcription factors in modulating placental responses to oxidative and inflammatory stress.

Downregulation of NRF1 observed in our study supports the hypothesis that mitochondrial biogenesis and energy homeostasis are impaired in preeclampsia. NRF1 is known to regulate the transcription of mitochondrial genes and coordinate oxidative phosphorylation [24,25]. Its suppression may result in defective ATP production and accumulation of reactive oxygen species (ROS), exacerbating trophoblastic injury and apoptosis. Previous studies have shown that silencing NRF1 leads to mitochondrial DNA instability and impaired trophoblast differentiation, both of which are characteristic features of preeclamptic placentas [26,27,28]. Therefore, diminished NRF1 expression could be a key molecular event linking hypoxia-induced mitochondrial dysfunction with placental insufficiency [29].

Conversely, the upregulation of NRF2 reflects an adaptive cellular response to oxidative stress. As a master regulator of the antioxidant response, NRF2 activates the transcription of detoxifying and cytoprotective genes [7,30,31]. The observed increase in NRF2 immunoreactivity in syncytiotrophoblasts, stromal cells, and endothelial structures suggests that the placenta attempts to counteract oxidative injury by enhancing antioxidant defenses. However, persistent oxidative stress in severe PE may overwhelm these compensatory mechanisms, leading to chronic inflammation and vascular injury [12,32]. This dual nature of NRF2—protective yet potentially maladaptive under prolonged stress—may explain the variable outcomes observed across different severities of PE [6,28].

The bioinformatics analyses performed in this study further clarified the molecular context of these findings. Downregulated NRF1-associated pathways were enriched in glutathione metabolism, HIF-1, and AMPK signaling, all essential for maintaining mitochondrial and metabolic balance [9,12,29]. These disruptions suggest that NRF1 suppression compromises both oxygen sensing and energy regulation. Meanwhile, NRF2-associated pathways were predominantly enriched in immune and inflammatory responses, including AGE–RAGE signaling, bacterial infection, and lipid metabolism [24,33]. Such enrichment indicates that NRF2 activation extends beyond antioxidant regulation, influencing inflammatory and immune networks that contribute to placental pathology [27,34].

Clinically, the opposing expression patterns of NRF1 and NRF2 provide insight into disease progression. Reduced NRF1 expression may serve as an indicator of impaired mitochondrial function, while elevated NRF2 may reflect compensatory stress adaptation [21,35]. The combination of these markers could thus represent a molecular signature correlating with disease severity. Moreover, our findings align with the “two-stage model” of preeclampsia, in which early placental hypoxia triggers oxidative and inflammatory cascades that drive systemic maternal symptoms [21,28,36].

Collectively, these findings suggest that NRF1 and NRF2 play distinct yet interconnected roles in the placental pathophysiology of preeclampsia. NRF1 suppression likely contributes to mitochondrial dysfunction and reduced cellular energy capacity, whereas NRF2 activation mediates antioxidant and immune responses aimed at mitigating oxidative injury. Their differential expression may therefore serve as a molecular indicator of disease severity and a potential therapeutic target for modulating oxidative stress in preeclampsia [37,38].

This study has several limitations. First, the analysis was limited to immunohistochemical assessment of NRF1 and NRF2 protein expression, without complementary quantitative assays such as Western blotting or qPCR to validate expression levels. Second, the cross sectional and single-center design restricts the ability to infer causal relationships or generalize findings to broader populations. Third, bioinformatic analyses were based on public databases and computational predictions rather than experimental validation, which may not fully capture tissue-specific molecular interactions in preeclampsia. Finally, clinical variables such as maternal comorbidities or medication use were not stratified in subgroup analyses, which could influence oxidative stress status. Future multicenter studies integrating molecular, genetic, and clinical parameters are needed to confirm and expand these findings.

## 5. Conclusions

This study demonstrated that NRF1 expression decreases while NRF2 expression increases in placentas from preeclamptic and severe preeclamptic pregnancies. These opposite changes suggest that impaired mitochondrial regulation through NRF1 and enhanced antioxidant defense via NRF2 together contribute to placental dysfunction in preeclampsia. Bioinformatic analyses supported these findings, linking NRF1 downregulation to metabolic and mitochondrial pathways, and NRF2 activation to oxidative and inflammatory responses. The combined evaluation of NRF1 and NRF2 may therefore provide insight into disease severity and reflect the balance between mitochondrial impairment and adaptive antioxidant responses. In conclusion, NRF1 and NRF2 represent promising molecular indicators of placental oxidative stress in preeclampsia and may serve as future targets for therapeutic modulation.

## Figures and Tables

**Table 1:** Demographics properties and laboratory parameters of patients.

Parameters	Control (*n* = 40)	PE (*n* = 40)	sPE PE (*n* = 40)	Multiple Comparison
Maternal age, year	28.5 ± 4.6	31.1 ± 5.3	31.8 ± 3.4	*p* > 0.005
Gravida, no unit	2.6 ± 1.2	2.4 ± 1.4	2.3 ± 1.2	*p* > 0.005
Parity, no unit	1.3 ± 1.2	1.1 ± 0.9	1.0 ± 0.8	*p* > 0.005
Systolic blood pressure, mmHg	111.2 ± 7.3	164.2 ± 15.4 *	175.3 ± 16.6 **	*p* < 0.05
Diastolic blood pressure, mmHg	65.7 ± 6.6	99.7 ± 10.35 *	111.4 ± 15.81 **	*p* < 0.05
24 h proteinuria output, mg/day	166.3 ± 65.2	1264 ± 322.4 *	1416 ± 450.5 **	*p* < 0.05
Alanine aminotransferase, U/L	15.4 ± 5.5	30.7 ± 12.6	34.8 ± 16.1	*p* > 0.005
Aspartate transaminase, U/L	23.5 ± 7.9	34.3 ± 11.1	35.1 ± 10.2	*p* > 0.005
Platelet count, 10^9^/L	284.4 ± 75.8	165.5 ± 68.1 *	147.4 ± 54.3 **	*p* < 0.05
Lactate dehydrogenase, U/L	305.3 ± 87.3	337 ± 99.6	325.5 ± 63.7	*p* > 0.005
Gestational week at birth, week	37.9 ± 1.42	34.1 ± 2.74	33.5 ± 1.95	*p* < 0.05
Fetal birth weight, kg	3320 ± 150.2	3014.5 ± 164.3 *	2700.6 ± 341.8 **	*p* < 0.05
APGAR score	9.2 ± 1.2	8.6 ± 1.6 *	8.1 ± 2.9 **	*p* < 0.05

PE: preeclampsia, sPE: severe PE, * control vs. PE: *p* < 0.01, ** control vs. sPE: *p* < 0.01.

## Data Availability

All data was presented in the study.

## Abbreviations

**Abbreviation**	**Full Term**
ACOG	American College of Obstetricians and Gynecologists
AGE–RAGE	Advanced Glycation End Products–Receptor for Advanced Glycation End Products
AMPK	AMP-Activated Protein Kinase
CS	Cesarean Section
DAB	Diaminobenzidine
EDTA	Ethyl Diamine Tetraacetic Acid
FDR	False Discovery Rate
GPX	Glutathione Peroxidase
HIF-1	Hypoxia-Inducible Factor 1
KEGG	Kyoto Encyclopedia of Genes and Genomes
NRF1	Nuclear Respiratory Factor 1
NRF2	Nuclear Factor Erythroid 2–Related Factor 2
NSD	Normal Spontaneous Delivery
PBS	Phosphate-Buffered Saline
PE	Preeclampsia
PPI	Protein–Protein Interaction
ROS	Reactive Oxygen Species
sPE	Severe preeclampsia
